# Circadian immunity from bench to bedside: a practical guide

**DOI:** 10.1172/JCI175706

**Published:** 2024-02-01

**Authors:** Huram Mok, Elaine Ostendorf, Alex Ganninger, Avi J. Adler, Guy Hazan, Jeffrey A. Haspel

**Affiliations:** 1 Division of Pulmonary and Critical Care Medicine, Department of Internal Medicine, Washington University School of Medicine, St. Louis, Missouri, USA.; 2 Department of Pediatrics, Soroka University Medical Center, Beer-Sheva, Israel.; 3 Research and Innovation Center, Saban Children’s Hospital, Beer-Sheva, Israel.

## Abstract

The immune system is built to counteract unpredictable threats, yet it relies on predictable cycles of activity to function properly. Daily rhythms in immune function are an expanding area of study, and many originate from a genetically based timekeeping mechanism known as the circadian clock. The challenge is how to harness these biological rhythms to improve medical interventions. Here, we review recent literature documenting how circadian clocks organize fundamental innate and adaptive immune activities, the immunologic consequences of circadian rhythm and sleep disruption, and persisting knowledge gaps in the field. We then consider the evidence linking circadian rhythms to vaccination, an important clinical realization of immune function. Finally, we discuss practical steps to translate circadian immunity to the patient’s bedside.

## Introduction

Circadian rhythms are daily oscillations in biological activity that help organisms adapt to the day-night cycle (1, 2). These rhythms originate from an internal molecular clock that provides an evolutionary and reproductive advantage (3, 4). As such, many aspects of human physiology are directly or indirectly under circadian control, including variations in blood pressure, metabolism, body temperature, and sleep timing (5). The immune system is no different. Studies documenting circadian rhythms in the development, distribution, and effector function of immune cells are increasing exponentially. However, the translation of such knowledge into effective clinical strategies is still in its infancy. Here, we review recent data on mechanistic connections between circadian rhythms and immunity, with an eye toward clinical application. To this end, we consider vaccination as a case study of how to use circadian immunity to optimize a medical intervention.

## Basic concepts

**Figure 1:**
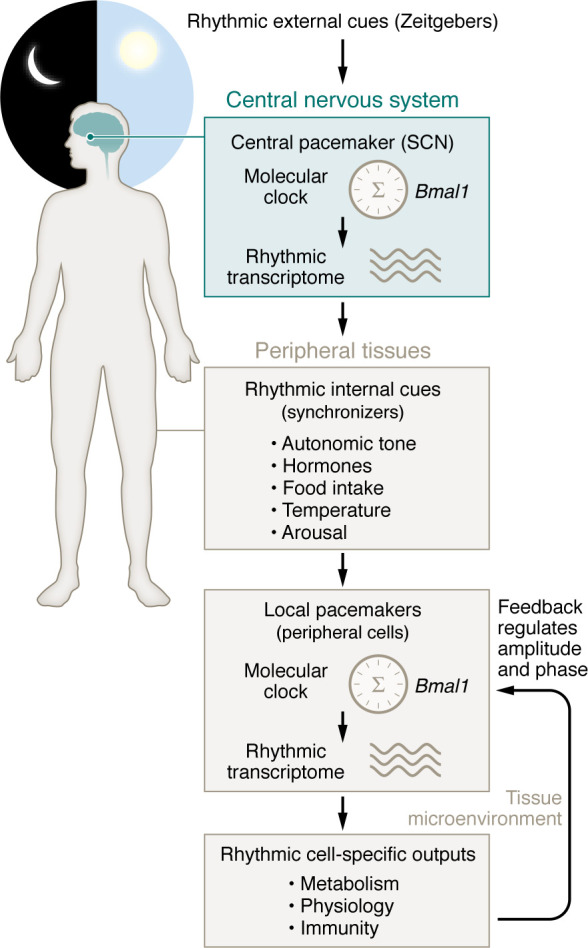
The oscillator model of circadian rhythm generation. In this model, the circadian molecular clock acts as a cell-autonomous rhythm generator (center) that produces rhythmic patterns of gene expression. At the organism level, the system has two parts: a central pacemaker housed in the central nervous system within the suprachiasmatic nucleus (SCN) of the hypothalamus and local pacemakers within peripheral tissues responsible for physiological outputs. To set the biological time of day (the circadian phase), the clock within the central pacemaker converts rhythms in external light into synchronized oscillations in hormone secretion (melatonin and hypothalamic pituitary axis), autonomic neural activity, arousal, appetite, and core body temperature. These rhythmic internal cues are converted by oscillators within peripheral cells into tissue-specific circadian rhythms. Circadian rhythms can modify the external environment around cells and, in so doing, can affect the amplitude and phase of the peripheral clock through feedback regulation.

Most of the daily patterns that we observe in organisms arise out of a combination of internally generated circadian rhythms plus extrinsic changes in the environment with the day-night cycle (6). The overall system that produces circadian rhythms is called an “oscillator model” (Figure 1). It has two parts: a central pacemaker housed in the CNS within the suprachiasmatic nucleus (SCN) of the hypothalamus and local pacemakers within peripheral tissues responsible for physiological outputs (7). Regardless of location, there is a self-contained rhythm generator or “clock” that has a roughly 24-hour-long cycle. The clock is synchronized to behavioral and environmental cues called zeitgebers that adjust its timing or phase by temporarily speeding the clock up (causing a phase advance) or slowing it down (causing a phase delay). The principal zeitgeber is light at the organism level, and the process by which a zeitgeber adjusts the phase of the clock is called entrainment. The clock imprints a daily rhythmic pattern on a wide variety of outputs at all levels of the biological scale, ranging from the abundance of small metabolites to physiological parameters like temperature and to behaviors like wakefulness (7). This includes standard clinical measurements of immune parameters (for example, white blood cell count, ref. 8) as well as the symptoms of inflammatory disorders, like asthma, and autoimmune disease (9). Some of the rhythmic outputs of the circadian system, such as temperature and cortisol variation, can themselves serve as internal synchronizing inputs (7). Such autoregulatory loops might enable the circadian system to adjust to the health status of the organism. For example, sleep timing is a circadian output where the system imposes daily rhythms in arousal (10, 11). However, sleep deprivation feeds back on the circadian system and alters circadian outputs in things like leukocyte gene expression (12, 13).

**Figure 2:**
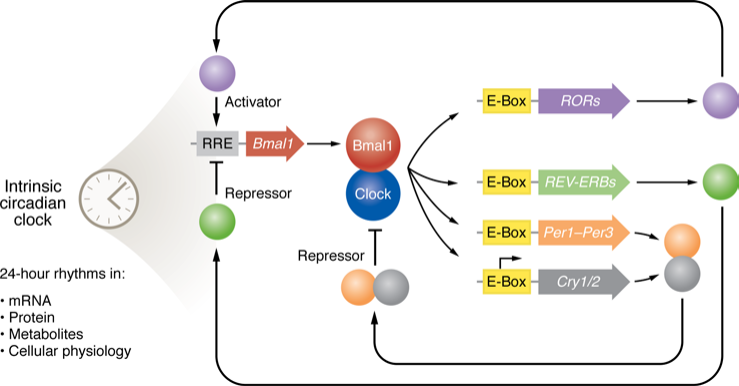
Schematic of the core molecular clock in mammals. The system consists of two fundamental loops, one providing negative feedback and the other providing positive feedback. Immune functions tied to specific molecular clock constituents are depicted. E-Box and ROR response elements (RRE) are depicted by yellow and gray rectangles, respectively. Coding sequences are depicted by large arrows, and proteins are depicted by circles. Note, this representation is simplified to demonstrate the basic feedback mechanism. Multiple accessory proteins either complex with core clock proteins or regulate clock gene expression, thereby providing stability and tuning to the system (151).

Our understanding of the circadian system has advanced greatly over the last 30 years, with the genetic dissection of the clock, first in *Drosophila*, later in mice, and, finally, validated in humans suffering from sleep-timing disorders (11, 14–16). In mammals, the clock is molecular in composition and conceptualized as a transcription factor network (Figure 2). At the center of the network is a transcription factor complex formed by the proteins BMAL1 and CLOCK that stimulate transcriptions at E-box promoter motifs (17). Targets of the BMAL1/CLOCK complex include other clock genes, such as *PER1–PER3*, *CRY1/2*, *NR1D1/2* (also called *REV-ERBa/b*), and *RORA-G* (18). Products of these genes feed back onto the core complex, thereby creating a daily periodicity in clock gene expression, often called the transcription-translation feedback loop (TTFL). Modifications of clock proteins like phosphorylation change their stability, function, and nuclear localization, thereby adjusting the speed (or period duration) of the TTFL and enabling zeitgebers to alter the phase of the clock (19).

Other targets of the molecular clock include transcription factors that add additional levels of regulation and have important downstream effector function in their own right, for example, nuclear factor IL-3–regulated (*NFIL3*), the PAR domain basic leucine zipper transcription factors (*DBP*, *TEF*, and *HLF*), and the peroxisome proliferator–activated receptors (*PPAR*s) (20–22). Altogether, the molecular clock imparts a daily rhythm to about 50% of protein coding transcripts organism wide (23, 24). Interestingly, while the core molecular clock is highly conserved across tissues, rhythmic gene expression downstream of the clock (called the circadian transcriptome) varies considerably from tissue to tissue (23). The power of the molecular clock as a model is that clock gene expression patterns can biochemically represent the time of day. In healthy individuals, clock gene expression in a tissue sample can predict with reasonable accuracy when during the day it was obtained (25–27).

Almost all nucleated cells express clock genes, including leukocytes and leukocyte progenitors (24). Thus, the immune system has molecular clocks embedded at the individual cell level, enabling it to incorporate temporal information into its metabolism, migration, and other key functions (2, 28). However, to coordinate clock-regulated functions in groups of leukocytes, as occurs during innate or adaptive immune responses, extracellular cues are needed to align the clocks of adjacent cells.

## Central and peripheral pacemakers

Under normal circumstances, synchronizing clocks in peripheral tissues is the job of the SCN in the ventral hypothalamus (29). This central pacemaker is synchronized by light information from the retina and innervates CNS nuclei regulating the autonomic nervous system, the hypothalamic-pituitary-adrenal axis, and the pineal gland, which produces melatonin (29–31). These systems, in turn, generate a variety of rhythmic cues within the organism, such as temperature, rest-arousal cycle, and nutrient abundance, that presumably work in combination to align individual cellular clocks in the periphery (29). There are also local factors that enforce circadian alignment between nearby cells, including extracellular matrix rigidity (32), cytokines like TGF-β that are stored in the extracellular matrix (33), and, intriguingly, type II cytokines (34).

Which cues are most important for immune cell clocks have not been fully defined. However, leukocyte trafficking into skeletal muscle depends on β-adrenergic signaling, suggesting CNS-driven rhythms in sympathetic tone to be important (35). Another study emphasized rhythms in glucocorticoid signaling as driving diurnal rhythms in T cell survival and homing to the spleen (36).

Desynchronization between the phase of central and peripheral clocks occurs when there is a sudden shift in the light/dark cycle, as occurs with jet lag. This happens because the central (SCN) clock adjusts to an abrupt change in light schedule very rapidly, whereas peripheral clocks adjust on the scale of days, depending on the magnitude of the light shift and factors like organism age (37). Desynchronization of central and peripheral clocks also can occur when zeitgebers are pitted in opposition, such as during night shift work, where nighttime activity and feeding schedule compete with SCN input (38). Like sleep deprivation, circadian misalignment is physiologically stressful and is marked by elevated IL-6 levels and shortened telomeres (39–43). What exactly circadian misalignment does to an organism’s health status is not fully clear. Elevated IL-6 levels by themselves have immunometabolic consequences, including enhancement of gluconeogenesis and immunosuppression (44). Other mechanisms may be at work; for example, jet lag enhances the homing of immunosuppressive Tregs to nascent tumors, thus contributing to tumor immune escape and promoting tumor growth (45). What is clear from epidemiological studies is that chronic circadian misalignment is associated with an elevated risk of various cancers, metabolic diseases, and infectious diseases like pneumonia (46–50). It is tempting to speculate that the immune effects of circadian misalignment could contribute to all of these.

## Limitations of the clock paradigm

The TTFL paradigm greatly advanced chronobiology research. However, it does have limitations that are worth considering. The genetic methods used to discover clock genes have engendered a gene expression–centric mindset around circadian rhythms. However, it is increasingly clear the transcriptional network that we call the molecular clock does not operate in isolation but rather is one cog in a broad-based system of rhythmic cellular physiology. This larger cellular context encompasses rhythms in metabolism (51–55), protein quality control (56, 57), signal transduction (58), and redox (59, 60), all influencing and reinforcing one another. As an example, alterations in mitochondrial function alter clock gene expression rhythms (53, 61). Red blood cells, which lack DNA and cannot perform transcription, have demonstrated cytosolic circadian oscillators in redox chemistry (59). The idea that the TTFL is the central repository of temporal information in a cell is probably too simple. Even when focusing solely on gene expression, the clock appears to produce very different circadian transcriptomes in different cell types or pathologic states (23, 51, 62). As a result, clock gene expression patterns tell us little about what the effector functions or outputs of the circadian system are in any given situation. The mechanisms that enable a conserved circadian clock to produce customized circadian transcriptomes are unclear. Part of the divergence between tissues may be an artifact of statistical underpowering or technical differences in rhythm detection algorithms (63, 64). Tissue-specific enhancers and chromatin accessibility likely play a part (65–67). Yet, there likely exist additional regulatory layers that connect the clock to specific downstream effectors based on cellular context, particularly in pathologic states. Because *Bmal1* is the sole clock gene whose deletion renders the molecular clock nonfunctional in mice, studies make frequent use of *Bmal1*-knockout models (68). However, *Bmal1* appears to have clock-independent functions, especially during embryonic development, and so phenotypes observed in *Bmal1*-deficient mice are not always due to circadian clock disruption (69).

Aging adds another layer of complexity. Changes to the circadian system in humans with aging are evident at multiple levels, ranging from altered sleep timing to changes in temperature regulation (70, 71). At a molecular level, aging is associated with reduced SCN activity, changes in hormone release profiles, and altered SCN and peripheral clock gene expression (72–79). The immune system undergoes changes with advanced age that affect activities associated with circadian rhythms, like phagocytosis and bactericidal activity (80). Leukocyte trafficking, which is highly rhythmic, becomes dysregulated with age, as do the expression patterns of cell-vascular adhesion molecules and cytokines (81). In addition, the adaptive immune responses, including vaccine responses, diminish with aging due to a reduction in lymphocyte production and affinity maturation (82). What this means is that the clinical effects of circadian immune rhythms ought to change with age. The key point is that is that the clock paradigm should not be overinterpreted as a proscriptive system that rigidly determines organism behavior. Rather, the clock provides temporal information to cells and organs around which their specialized functions can be organized in context.

## Circadian rhythms and the immune system

Because the immune system is distributed across the body, three sets of circadian clocks influence its function. First, there is the cell-autonomous molecular clock within each leukocyte, which is able to regulate the expression of cell surface molecules and signaling. Second, there is the central pacemaker in the CNS that controls synchronizing neurohormonal and behavioral cues. Finally, there are cellular clocks native to organs and structures that the leukocyte is moving through, which are able to regulate vascular adhesion, extravasation, and transcellular biosynthetic processes requiring leukocytes, like eicosanoid production.

### *Rhythms in basic immune processes*.

The recognition that circadian rhythms and the immune system are connected began with the observation that blood lymphocyte counts vary with the time of day (83). These observations now extend to rhythms in virtually all levels of fundamental immune function, including leukocyte development, trafficking, and cell-specific effector function (Table 1 and Table 2). At the basic research level, most studies make use of clock gene–deficient mice to support a direct circadian clock role in each observation. Other research highlights the immunological effects of environmental circadian misalignment in chronic inflammatory disease models. For example, mice exposed to jet lag exhibited higher gut barrier permeability, altered microbiota composition in favor of harmful bacteria, and chronic inflammation (84, 85). A similar phenotype could be produced using non-jet-lagged *Bmal1*-knockout mice (86, 87). In melanoma and breast cancer tumor models, jet-lagged mice showed increased tumor growth and accumulation of myeloid-derived suppressor cells that facilitate immune evasion (88). In the hippocampus, jet lag was correlated with suppressed *Il1b* and *Nfkbia*, though the significance of this observation is yet to be determined (89). While experimental jet lag studies are less specific than genetic ablation of clock genes, they may be more clinically relevant, as 4%–20% of the population in economically developed countries engages in some degree of night shift work (90, 91). They also help to explain growing epidemiological evidence linking low circadian system robustness (usually judged by actigraphy or sleep fragmentation) to hospitalizations for a variety of ailments (92). For example, in inflammatory bowel disease circadian misalignment correlates with a more aggressive course of disease, increased intestinal permeability, elevated blood TNF-α, and elevated stool calprotectin levels, which are considered indicative of neutrophil migration and inflammation of the intestine (93). Conversely, clock gene expression is significantly reduced in patients with active ulcerative colitis compared with that in healthy controls and those in remission, suggesting that feedback regulation of intestinal circadian clocks might contribute to pathogenesis (94).

### *Crosstalk with sleep*.

While numerous studies address how abnormal sleep affects immune parameters, differences in study design limit one’s ability to draw precise conclusions from this body of literature (95, 96). In general, interventions intended to disrupt sleep acutely or chronically alter immunological parameters like circadian disruption. For example, acute sleep deprivation reduces circulating leukocyte counts, alters systemic cytokine production, and compromises NK cell activity (96–99). Recent transcriptomic analysis of lungs from sleep-deprived mice showed downregulation of multiple genes that regulate immunity, such as NF-κB signaling regulators, chemokine receptors, and leukocyte migration, while transcripts that promote viral replication and immune evasion were enhanced (100). A separate study showed that *Bmal1* deficiency and sleep suppression were associated with decreased monocyte trafficking to the circulation (101). Monocytes and neutrophils from sleep-deprived mice had enhanced ROS production (101). The fact that circadian and sleep disruption converge toward similar phenotypes is not surprising because these activities are inevitably intertwined under naturalistic conditions. It is often difficult to determine whether some biological rhythms are directly driven by the molecular clock or indirectly through the sleep/wake cycle (which is influenced by the clock). In practical terms, the formal origins of immunologic biological rhythms are probably less important than their biological and clinical impacts.

### *Modifying factors*.

Evidence linking aging to circadian clock function in immune effector cells has emerged recently. In peritoneal macrophages obtained from aged mice, rhythms in macrophage trafficking, phagocytic activity, and the circadian transcriptome are strongly suppressed (102). Remarkably, clock gene expression in these cells is nearly identical to that in cells from young mice, emphasizing the pitfalls of solely relying on the TTFL to infer circadian system function (102). The relationship between chronic jet lag and immune senescence was explored as well. Mice subjected to chronic jet lag had shorter life spans and activation of inflammation-related pathways on transcriptomic analysis. Flow cytometry of the spleen and mesenteric lymph nodes showed an increased senescent T cell profile, indicated by higher frequencies of senescence-associated T cells, T follicular helper cells, and Tregs (103).

To summarize, there is now vast literature documenting biological rhythms in the function of individual immune cells and collective immune responses, much of which has been mechanistically tied to the molecular circadian clock. But how does this fundamental biology apply to important clinical applications of immunology? Below we consider the case of vaccination.

## Circadian rhythms in the vaccination process

Vaccines are one of the most consequential medical interventions ever devised. While the science and art of vaccinology continue to advance, as illustrated during the COVID-19 pandemic, the fundamentals of the vaccine response are now well understood (104). Vaccination begins at the site of entry, with internalization of the immunogen by dendritic or other antigen-presenting cells. Efficient processing of the immunogen requires DC activation through an innate inflammatory stimulus that in most vaccines is generated by a chemical adjuvant. DCs then traffic to local area lymph nodes, where they present antigens derived from proteolytic processing of the immunogen in the context of cell surface major histocompatibility type II (MHC-II) complexes. In this way, DCs initiate the selection and differentiation of antigen-specific B and T cells. The B cell effector arm is thought to confer dominant clinical protection from vaccines as neutralizing antibodies can break the chain of infection. The role of antigen-specific CD8^^+^^ T cells is less well understood, but these likely are important for the clearance of cells infected with obligate intracellular pathogens like viruses. The final step in the vaccination response is the induction of long-term immunologic memory through the differentiation of specialized B and T cells.

**Figure 3:**
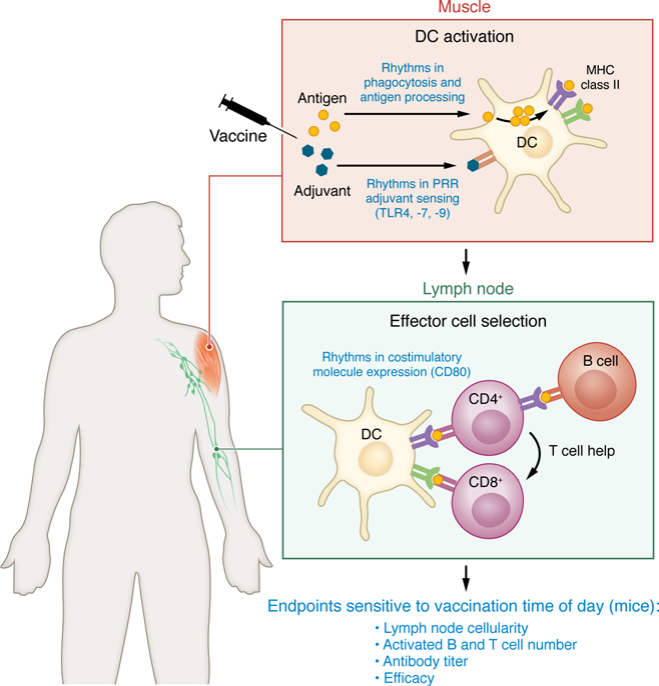
Circadian regulation of the vaccination process. Syntheses of studies conducted in mice that examine clock regulation of immune processes that contribute to vaccination are described in blue text. These include DC activation at the site of intramuscular injection (105, 106, 152), trafficking of DCs and lymphocytes to the lymph node (107, 153, 154), selection of antigen-specific T and B cells (108, 109, 112), and effector function (111, 112).

Circadian regulation appears to be present at most of the steps in the vaccination process (Figure 3), beginning with the adjuvant. Many adjuvants activate innate inflammation by engaging TLRs like TLR4 and TLR9 that have circadian rhythms in expression (105). Using the TLR9 agonist CpG DNA as an adjuvant, Silver et al. showed that vaccine responses can be optimized by immunizing mice at the time of day when the key adjuvant receptor is maximally expressed (105). The next step in vaccination, antigen processing by the DC, also has a circadian rhythm that is lost in *Bmal1*-knockout cells in mice (106). In this case, the mechanism has been attributed to circadian variations in mitochondrial morphology and calcium content within the DC (106). DC trafficking to the lymph node is yet another point of circadian clock control. Here, rhythmic trafficking requires the coordination of molecular clocks residing in DCs and lymphatic endothelial cells (107). The result is the synchronized rhythms of cell adhesion molecules and their ligands on the dendritic and endothelial cell surfaces as well as the chemoattractant CCL21 (107). Finally, the time of day that DCs arrive in the lymph nodes also seems to matter in terms of the amount of antigen-specific CD8^^+^^ T cell proliferation induced (108, 109). This effect requires genetically intact molecular clocks in both DCs and T cells (110), resulting in greater numbers of DC and T cells within the lymph node and rhythmic expression of the costimulatory molecule CD80 on DCs (111). Recently, Ince et al. tried to address the question of what all these layers of control amount to in terms of the best and worst times to vaccinate (112). Using a combination of observations in mice and mathematical modeling, they suggest most of the above layers of circadian regulation yield maximal immune responses when vaccination is initiated midday in mice (112). The effect of optimal vaccination timing was long lived, in that daytime vaccination produced higher antibody titers, antigen-specific CD8^^+^^ T cells, and memory cells 28 days after immunization (112). Counterintuitively, the best time to immunize mice proved to be daytime, which corresponds to their rest period when contact with infectious agents ought to be the lowest. This may conceivably be the case with humans as well: there are little data on the utility of late-night vaccinations in patients. On the other hand, the optimal timing observed in mice might be an adaptation of inbred strains that have been cohoused for numerous generations and, unlike feral mice, nest together while sleeping. In such a circumstance, enhanced immunity during the rest phase might limit horizontal transmission of infection. An additional consideration is that mice used in standard research conditions are housed in clean barrier facilities, leading to a lack of immune experience and variable colonization with commensal organisms that might affect immune circadian rhythms (113). Moreover, the usual practice of housing research animals at temperatures comfortable for humans produces cold stress in mice and this may confound immune readouts (113, 114). Nevertheless, studies conducted in mice point to layers of circadian control of vaccine responses. These studies stop short of testing the efficacy of timed vaccination. For this, one must consider emerging clinical literature.

## Clinical evidence for rhythms in vaccine performance

Basic research studies document diurnal variations in immune responses to antigenic challenges. However, finding this signal in clinical settings is challenging. People have diverse lifestyles, are frequently exposed to light at night, engage in night shift work, and have differing activity patterns on the weekend versus the weekday, all of which can affect the phase of their circadian rhythms. Due to cost, prospective clinical studies are limited in terms of sample size, diversity, and follow-up. Against this backdrop, a handful of studies investigated whether the time of vaccine administration affects immune responses in humans, typically using outpatient clinic populations or defined cohorts (e.g., healthcare workers) as samples of convenience. While some reports supported morning-time vaccination as optimal, others found no difference between morning and afternoon vaccination (115–120). One report favored the afternoon (121). A recent trial comparing morning and afternoon influenza vaccination showed no differences in antibody titer between the two groups, but subgroup analysis weakly favored morning vaccination in adults over 65 and women (122). Limited number of participants and heterogeneity of vaccines studied (influenza, hepatitis B, equine encephalomyelitis virus, TB) likely led to mixed results from these studies. Importantly, it was impractical in these studies to test vaccine efficacy (i.e., the degree of clinical protection), and they employed vaccine-elicited antibody titers as the primary endpoint. The problem with antibody titers is that there is large intersubject variability, even among healthy individuals (123), and it has proved a poor surrogate of vaccine efficacy (124). Moreover, research in mice indicates that antibody titers are only mildly affected by the time of vaccination even under highly controlled conditions, making it a relatively insensitive marker of circadian effects (112).

The outbreak of COVID-19 brought on a global public health crisis. To meet this challenge, multiple vaccines were developed in a short period, and strategies to optimize vaccine responses gained a renewed focus (Table 3). Of these, several studies examined the effect of SARS-CoV-2 vaccination timing on immune response through antibody titer measurements, with conflicting results. One manuscript supported the superiority of morning vaccination, two of them afternoon, and no significant differences were seen in the rest (125–129). These studies, similar to their predecessors, were (a) limited by small sample sizes, in which the largest cohort was 2,800; (b) aimed at a specific group such as healthcare workers or university students; and (c) variable in vaccine types and doses (125–129). Most importantly, conclusions were based largely on antibody titers, with attendant limitations. The question of how sleep affects vaccine responses is similarly hamstrung. A recent meta-analysis reviewed 7 studies in which participants were subjected to sleep deprivation before being immunized, with a combined sample of 304 patients receiving influenza, hepatitis A, or hepatitis B virus vaccines (130). While antibody titers in experimentally sleep-deprived patients were lower, what this translates to in terms of clinical protection is unclear.

An alternative approach to investigating biological rhythms in vaccination is to leverage big data. In a recent study, our group used electronic medical records (EMRs) from a large HMO in Israel to examine associations between COVID-19 vaccine effectiveness and the time of day vaccination occurred (131). The idea was that population-level data (1.5 million patients in this case) would help overcome limited statistical power and enable a measurement of real-world vaccine effectiveness, allowing us to avoid relying on surrogate markers. It further allowed a higher resolution of data analysis beyond arbitrarily defined bins like “morning” versus “evening.” What made the study work was that Israel has a national EMR database that tracks all medical encounters, and the Ministry of Health strongly incentivized official COVID-19 testing, so there was minimal censoring. The data indicated that late-morning to early-afternoon COVID-19 vaccination was associated with fewer breakthrough infections (131). This effect was primarily driven by benefits in the younger (<20 years old) and older (>50 years old) demographics. Another recent study used a large database of deidentified patient data to examine associations between COVID-19 breakthrough infections and sleep disorders (132). They analyzed a cohort of roughly 24,000 patients with a sleep disorder diagnosis (98% insomnia, 4.5% with circadian rhythm sleep disorder) who received at least 2 doses of the Pfizer or Moderna-produced mRNA vaccines. Compared with propensity-matched controls, a sleep disorder diagnosis was positively associated with COVID-19 breakthrough infection (132). Thus, population-level databases can detect clinically important relationships among vaccination, biological rhythms, and sleep. They enable one to identify who within a population would benefit from modifying healthcare delivery to optimize sleep and circadian factors and to what degree.

## Moving circadian immunity from bench to bedside

Circadian and sleep biology have the potential to make existing medical interventions better without increasing costs to patients or the healthcare system (133). However, modern civilization is in a sense designed to free people from a rigid day-night cycle, and neither patients nor caregivers are likely to embrace interventions that they find proscriptive. How then can we successfully deploy circadian regulation of immune function in clinical practice? A review of current literature offers some practical suggestions.

### Focus on controlled medical settings.

There is compelling evidence that the therapeutic index of many popular medications varies with time of day, including immunomodulatory drugs like corticosteroids (134). In the real world though, patients frequently do not take medications as prescribed (135). Therefore, it makes sense to focus on chronotherapy interventions given in controlled settings. Such settings include inpatient wards, infusion centers, surgeries, and outpatient clinics where time of sample collection and medical interventions can be tracked (6). As examples, checkpoint inhibitors given for metastatic melanoma or R-CHOP chemotherapy given for B cell lymphoma appear to be more efficacious when given in the afternoon to early evening (136, 137). A future opportunity in this space may be to establish the optimal timing of corticosteroids that are part of a great many chemotherapy regimens. One mouse-based study found that glucocorticoid signaling at night stimulates T cell survival via IL-7 receptor signaling (36). This opens the possibility that corticosteroids could increase antitumor immunity if given at the right time of day.

### Focus on public health benefits.

Because of interindividual differences, applying circadian biology to individual outpatients would ideally involve knowing the phase of their relevant circadian rhythms in real-time. However, currently, we do not have the technology to accomplish this at scale, although various groups are developing wearable or implantable devices (138–140). Even if we had the technology, and the predictor models of circadian phase that the technology would rely on were perfectly accurate, there is a limit to how precisely in time clinical interventions can be delivered in real-world settings. While a four-hour interval of the day could reasonably be targeted to provide a treatment in standard clinical practice (targeting, say, a single hour of the day), achieving a benefit from biological rhythms is infeasible short of an automated implant (like an insulin pump). For now, public health interventions like mass vaccination campaigns are good targets for chronotherapy, because, at this scale, interindividual differences will average out and only a moderate level of temporal precision appears to be required (about 4 hours) (131). While the effect size of circadian-timed vaccinations applied based on population norms would likely be smaller than the effect of individualized therapy, extending circadian-timed vaccinations over thousands or millions of patients would amount to a large aggregate benefit. Another possible avenue is public policy. Recently, a campaign in the US Congress to legislate permanent daylight savings time brought to the fore research on the detrimental effects of moving the clocks ahead one hour in the spring, essentially a short period of jet lag (141). We know of no studies examining the immunological effects of moving the clocks ahead, but this seems biologically plausible. The sleep and circadian biology research community favors permanent standard time as an alternative, as this would maximize activity during daylight hours in the winter, especially for schoolchildren (142–145).

### Concrete clinical endpoints.

For biological rhythms concepts to be embraced by patients and clinicians, the benefits must be tangible and seen as worth the effort. To this end, studies looking to apply circadian medicine should be designed and powered whenever possible to assess clinically important endpoints in addition to biomarkers. For example, one recent chronotherapy trial examined the administration of total parenteral nutrition in pediatric bone marrow transplant recipients, comparing 20- to 24-hour-a-day feeding (the current standard of care) with daytime-restricted feeding (146). Time-restricted feeding is a nutrition strategy in which caloric intake is limited to a period of 10–12 hours. Time-restricted feeding has shown to improve metabolic diseases in animal models, such as obesity, glucose intolerance, and dyslipidemia (147). The rationale for the trial was to test whether a feeding strategy consonant with normal circadian rhythms would be beneficial in humans. They found that daytime-restricted feeding was associated with a faster transition to an oral diet and a shorter hospital stay (146).

### Identify and target patient subsets that derive the most benefit.

Taking COVID-19 vaccination as an example, if every patient benefited equally from receiving COVID-19 immunization within the same narrow time frame, information regarding timing would be clinically useless. This is because immunizing an entire population within a narrow time frame would be at odds with attaining herd immunity in the shortest possible time. For chronobiology to improve public health, it will be necessary to identify the subsets of patients who should be prioritized for dosing at biologically optimal times of day. In the case of our recent study and others, timed vaccination seemed to disproportionately benefit older patients (116, 122, 131). Prioritizing older patients for vaccines earlier in the day would make it possible to derive clinical benefits from circadian-timed dosing without impeding vaccine uptake for the general population. In a similar vein, administering R-CHOP chemotherapy in the afternoon to patients with B cell lymphoma appears to primarily benefit women. Chemotherapy infusion centers could realize the benefits of this biological rhythm for patients without impeding their workflow simply by scheduling female patients for later in the day.

### Capitalize on big data.

A key lesson of the last few years is that large, high-quality data sets can be a major asset in medical research that approaches the utility of randomized controlled trials. Longitudinal cohorts, such as the UK Biobank and data sets collected during the COVID-19 pandemic, have helped to unmask the real-world contributions of biological rhythms and sleep in disease. The challenge is how to obtain this kind of data more widely in the future. Studies routinely capitalize on local EMR systems to observe biological rhythms in disease activity (e.g., asthma exacerbations, ref. 148) or in the processes of care (like inpatient medication dosing, ref. 149). However, the full power of such data to detect optimal times of day with precision is only realized at population-level scales. While EMRs are mandated in the US, the lack of a unified system fragments data collection, and there are multiple proprietary EMR products in use. That said, some EMR platforms, like EPIC and Cerner, have achieved significant market share nationwide and have “care everywhere” functions that allow the extraction of patient data across various hospitals using their platform. It would be extremely valuable to have a central research interface that could distill existing EMR data networks into useful deidentified data sets, perhaps administered and maintained by a responsible government body such as the NIH or the CDC. An alternative private enterprise solution to advancing circadian medicine would be the use of synthetic databases for research purposes (150). These databases contain large amounts of well-annotated patient data that are deidentified by introducing small random variations in the information recorded. To use these sources for analyzing biological rhythms in immunity or other clinical aspects, it would be necessary to decode specific fields so they accurately represent the original time stamps. A mechanism to obtain special permission for such decoding from either private or public entities would be extremely valuable in translating biological rhythms and sleep research to the clinic.

## Concluding remarks

This is an exciting time for biological rhythms research. As connections between circadian rhythms and immunity continue to emerge from basic research, there is a growing capacity to translate this knowledge into clinical settings. For all the effort that has gone into understanding circadian immunity, figuring out what times of day medications or vaccines should be given may not sound like much. In fact, it represents a sea change in the process of care. Most healthcare processes today are designed around what is most efficient for providers rather than the inherent biology of the patient. In this respect, chronotherapy represents a break from the past and a philosophy that fundamental biology should inform the provision of care for patients, not just drug design.

## Figures and Tables

**Table 3:**
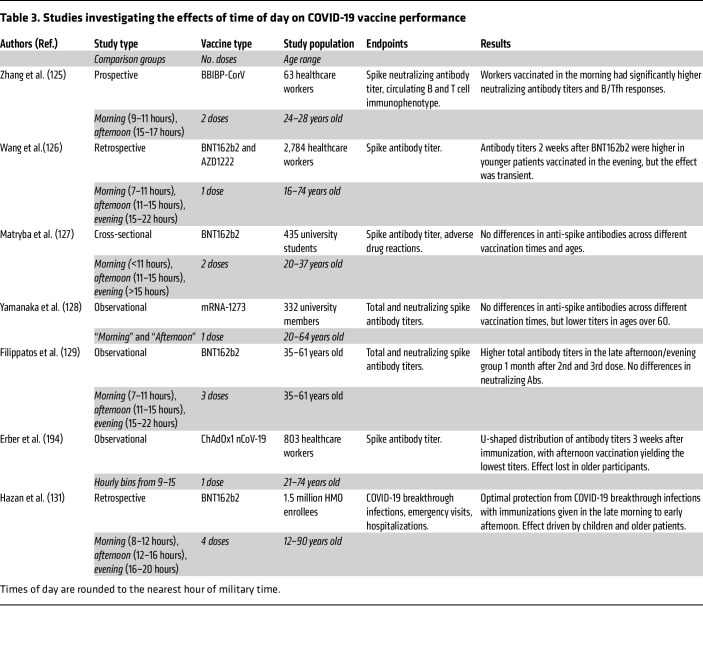
Studies investigating the effects of time of day on COVID-19 vaccine performance

**Table 2:**
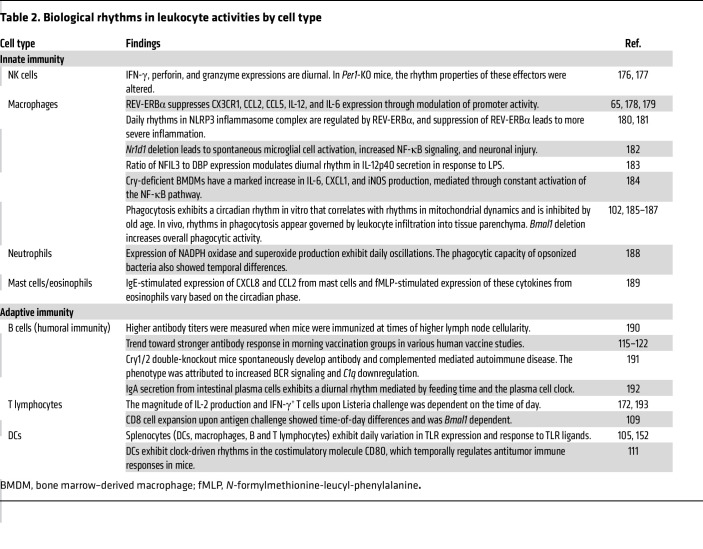
Biological rhythms in leukocyte activities by cell type

**Table 1:**
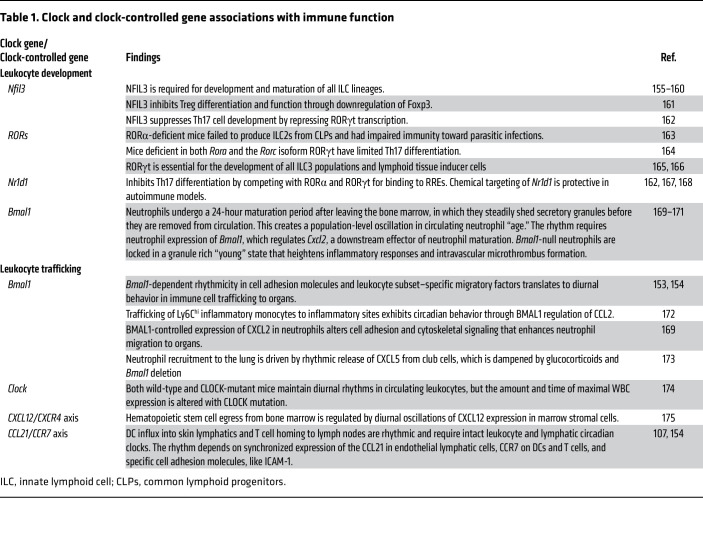
Clock and clock-controlled gene associations with immune function

## Version 1 02/01/2024

Electronic publication
